# Hospital Surgical Volume and Regional Disparities in Congenital Heart Surgery Outcomes: Analysis of Korean National Health Insurance Claims Data, 2002–2021

**DOI:** 10.3390/medicina62020355

**Published:** 2026-02-11

**Authors:** Ji-Sook Kim, Hyeong-taek Woo, Jong-Yeon Kim, Hang-Me Nam, Hye-Jin Lee

**Affiliations:** 1 Department of Pediatrics, School of Medicine, Kyungpook National University, Daegu 41944, Republic of Korea; jisook.kim.neo@gmail.com; 2 Department of Preventive Medicine, Keimyung University School of Medicine, Daegu 42601, Republic of Korea; 3 Department of Preventive Medicine, School of Medicine, Kyungpook National University, Daegu 41944, Republic of Korea; kom824@knu.ac.kr; 4 Daegu Public Health Policy Institute, Daegu 41940, Republic of Korea; kjd1213@nate.com (H.-M.N.); garden_1@hanmail.net (H.-J.L.)

**Keywords:** congenital heart surgery, hospital volume, regional disparities, J-STAT, Korea

## Abstract

*Background and Objectives*: The volume–outcome relationship in congenital heart surgery (CHS) has been widely reported internationally, but systematic nationwide evidence from Korea remains limited. Given the concentration of high-volume centers in the Seoul Capital Area (SCA), we aimed to examine whether hospital surgical volume was associated with short-term mortality and to what extent regional disparities could be explained by differences in surgical volume. *Materials and Methods*: We conducted a nationwide retrospective cohort study of 31,150 patients who underwent CHS in 91 hospitals in Korea between 2002 and 2021 using National Health Insurance claims data. Hospitals were classified by location (SCA vs. non-SCA). Annual surgical volume was defined using two approaches, (i) above vs. below the overall mean annual volume (17.1 cases per hospital), and (ii) three categories (≤20, 21–40, and >40 cases/year). The primary outcome was 30-day mortality. Multivariable logistic regression adjusted for case mix, including J-STAT category, sex, hospital type, age, prematurity, and low birth weight. Hospital-level variation was further evaluated using generalized linear mixed models with random hospital intercepts, and intraclass correlation coefficients (ICCs) were estimated to quantify between-hospital variation and the explanatory contribution of surgical volume. *Results*: Overall 30-day mortality was 1.99%, with higher mortality observed in non-SCA hospitals compared with SCA hospitals (3.19% vs. 1.57%). After adjustment, lower annual surgical volume was strongly associated with higher 30-day mortality. Compared with hospitals performing >40 cases/year, the adjusted odds ratios were 4.13 (95% CI, 3.30–5.17) for hospitals performing 21–40 cases/year and 4.95 (95% CI, 3.98–5.95) for those performing ≤20 cases/year. In multilevel analyses, annual surgical volume accounted for 54% of the between-hospital variation in 30-day mortality. Adjustment for surgical volume substantially attenuated the regional disparity, with the odds ratio for non-SCA versus SCA hospitals decreasing from 2.12 (95% CI, 1.80–2.49) to 1.14 (95% CI, 0.95–1.37). *Conclusions*: A strong volume–outcome relationship exists in congenital heart surgery in Korea, with excess mortality concentrated in low-volume hospitals rather than regional location itself. Regional disparities in outcomes appear largely attributable to the uneven distribution of surgical volume. Strategies focused on service consolidation and strengthened referral to high-volume centers may be effective in reducing inter-hospital variation and improving national outcomes in pediatric cardiac surgery.

## 1. Introduction

Globally, numerous studies have investigated the relationship between hospital surgical volume and outcomes in congenital heart surgery (CHS), and it is now well established that higher surgical volume is associated with improved postoperative survival and reduced morbidity [1,2,3,4,5,6,7,8,9,10,11]. Reflecting this evidence, the 2023 U.S. consensus statement for pediatric heart surgery centers, developed by the Society of Thoracic Surgeons (STS) and collaborating societies, recommended that comprehensive centers perform at least 200 congenital heart operations/year to maintain quality and team competence [12]. In Korea, where healthcare resources and patient referrals are highly concentrated in the Seoul Capital Area (SCA), most high-volume congenital heart surgery centers are located in this region [13,14,15,16]. This concentration of surgical capacity may lead to regional differences in access to care and postoperative outcomes among children with congenital heart disease. To the best of our knowledge, only one study has examined the relationship between hospital surgical volume and outcomes of congenital heart surgery in Korea. Using data from the Korea Heart Foundation covering 2000–2014, the study provided valuable descriptive insights but was limited by dated data, insufficient adjustment for case mix, and the absence of regional information, as hospitals were categorized solely by surgical volume without considering geographic context [17]. Therefore, using the nationwide National Health Insurance Service (NHIS) claims database, we investigated whether outcomes of congenital heart surgery among patients diagnosed with congenital heart disease who underwent surgery before the age of 18 differ by hospital surgical volume and region in Korea, after adjusting for case mix, thereby providing contemporary national evidence and addressing a major gap in the current literature.

## 2. Materials and Methods

### 2.1. Data Sources and Study Sample

This study was conducted using customized data from the Korean National Health Insurance Service (NHIS) claims database, which provides de-identified, individual-level information on healthcare utilization, diagnoses, and procedures. The NHIS database is compiled from insurance claims submitted by healthcare providers to the NHIS after delivering covered medical services, and de-identified versions of these data are made available for research purposes [18]. We constructed a customized dataset by identifying individuals who received medical care between 2002 and 2021 for perinatal conditions (codes beginning with P) or for congenital malformations, deformations, and chromosomal abnormalities (codes beginning with Q), as defined in the Korean Standard Classification of Diseases (KCD), which is derived from the International Classification of Diseases (ICD). Using this customized dataset, we further identified patients who underwent congenital heart surgery by selecting those with diagnostic codes for congenital malformations of the circulatory system (KCD codes beginning with Q2) and corresponding cardiac surgical procedures defined by the authors based on previous studies [19] (Supplementary Table S1). Among these patients, individuals who were born before 2002 or received their first congenital heart surgery after 18 years of age were excluded from the analysis.

### 2.2. Outcomes

The primary outcome was all-cause mortality within 30 days after surgery. Mortality was ascertained through linkage to the national death registry (Statistics Korea). We also evaluated all-cause mortality within 90 days after surgery as a sensitivity analysis. We first examined the association between hospital surgical volume and 30-day mortality in the overall cohort. Surgical volume was assessed in two ways: (1) above versus below the overall mean annual volume of 17.1 cases per hospital/year over the study period, and (2) categorized into three ordered annual volume groups to reflect low, medium, and high surgical volume: low-volume hospitals performed ≤20 cases/year, medium-volume hospitals performed 21–40 cases/year, and high-volume hospitals performed >40 cases/year. The volume–outcome relationship was further evaluated across Japanese Surgical Risk Categories (J-STAT) to assess consistency by case complexity [7,20]. J-STAT is an adaptation of the Society of Thoracic Surgeons-European Association for Cardio-Thoracic Surgery (STAT) mortality categories for Japanese CHS data. No Korea-specific risk-adjustment system suitable for nationwide claims data is currently available. We therefore used the J-STAT mortality risk categories, which were developed and validated in an East Asian setting, as a pragmatic approach to stratify baseline risk. Subsequently, hospitals were stratified by geographic location (SCA vs. non-SCA), and the same analyses were repeated to determine whether the volume–outcome pattern and mortality risk differed between hospitals located in and outside Seoul. For the 90-day sensitivity analysis, we focused on (1) whether the volume–outcome association persisted in the overall cohort and (2) whether adjustment for annual surgical volume attenuated the mortality difference between non-SCA and SCA hospitals (Tables S3 and S4).

### 2.3. Statistical Analysis

Baseline characteristics were compared using the chi-square test. We evaluated the association between hospital surgical volume and 30-day mortality after congenital heart surgery using a structured modeling sequence: (1) multivariable logistic regression for risk adjustment and estimation of overall associations, followed by (2) sequential multilevel (mixed-effects) logistic regression models with hospital-level random intercepts to account for clustering of patients within hospitals and to quantify between-hospital variation. In the multivariable logistic regression, we adjusted for J-STAT risk category, sex, hospital type (tertiary, secondary, or primary), prematurity, low birth weight, and age. The *p* for trend was obtained by assigning ordinal scores to the three annual volume groups and entering this variable as a continuous term in the logistic regression model.

For multilevel analyses, patients were the unit of analysis and 30-day mortality was the dependent variable. The base model included patient-level covariates only; the +Capital model additionally included hospital location (Seoul Capital Area [SCA] vs. non-SCA); and the +Capital + Volume model further included hospital surgical volume as a second-level predictor. Hospital volume was treated as the primary exposure of interest, whereas the regional indicator (SCA vs. non-SCA) was included to support interpretation from an equity/access perspective. Intraclass correlation coefficients (ICCs) were calculated for each model, and the proportion of between-hospital variation explained was derived as 1 − (ICC_model/ICC_reference), using the base model as the reference for “% explained vs. Base” and the +Capital model as the reference for “% explained vs. Capital” (i.e., the incremental contribution of volume). *p* values for added hospital-level variables were obtained from likelihood ratio tests comparing nested multilevel models (Base vs. +Capital; and +Capital vs. +Capital + Volume). All statistical analyses were performed using SAS Enterprise Guide version 7.1 (SAS Institute Inc., Cary, NC, USA).

## 3. Results

### 3.1. Study Population and Baseline Characteristics

**Figure 1:**
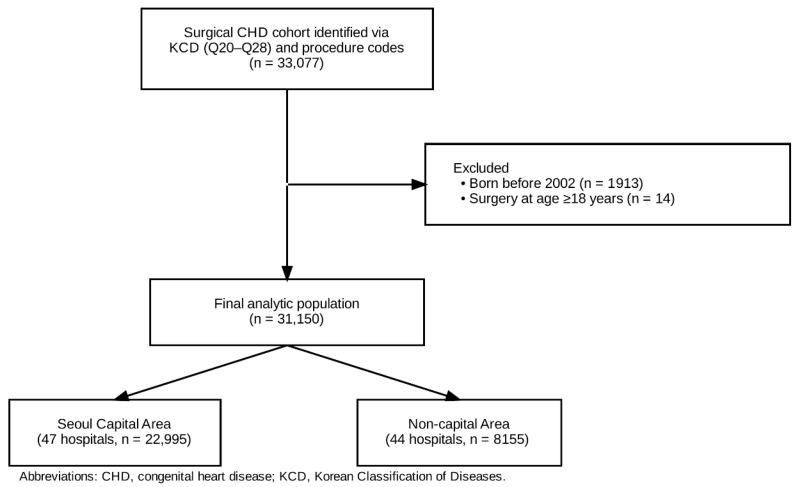
Flowchart of the study population selection process.

As shown in Figure 1, we identified 33,077 patients who underwent surgery for congenital heart disease using KCD diagnosis codes (Q20–Q28) and corresponding procedure codes. We excluded patients born before 2002 (n = 1913) and those who underwent surgery at age ≥ 18 years (n = 14), yielding a final analytic cohort of 31,150 patients. These patients were treated across 91 hospitals (SCA, 47 hospitals; n = 22,995; non-SCA, 44 hospitals; n = 8155). Table 1 summarizes patient characteristics by hospital region. Sex distribution was similar between regions (male, 52.6% vs. 51.9%; *p* = 0.2901). Compared with SCA hospitals, non-SCA hospitals treated a higher proportion of infants aged <1 year (67.7% vs. 63.1%; *p* < 0.0001) and had a greater proportion of patients born preterm (<37 weeks; 16.9% vs. 12.4%; *p* < 0.0001) and with low birth weight (<2500 g; 13.2% vs. 9.2%; *p* < 0.0001). Case-mix also differed by J-STAT category (*p* < 0.0001), with non-SCA hospitals having a higher proportion of J-STAT 2 cases (45.3% vs. 36.4%) and a lower proportion of J-STAT 3 cases (13.3% vs. 20.6%) than SCA hospitals. Table 2 presents hospital-level volume characteristics by region. Across all 91 hospitals, the total 20-year surgical volume was 342 cases per hospital (range, 1–6283), corresponding to a mean annual volume of 17.1 cases/year (342/20). SCA hospitals had substantially higher volumes than non-SCA hospitals (489 [range, 1–6283] vs. 185 [range, 1–2284]). When hospitals were classified by mean annual volume, the majority were low-volume (≤17.1 cases/year) in both regions (83.0% in SCA vs. 88.6% in non-SCA), whereas only 14.3% of hospitals overall exceeded 17.1 cases/year (17.0% in SCA vs. 11.4% in non-SCA). Using the three-tier classification, 85.7% of hospitals performed ≤20 cases/year, 5.5% performed 21–40 cases/year, and 8.8% performed >40 cases/year; the proportion of high-volume hospitals (>40 cases/year) was higher in SCA than in non-SCA regions (10.6% vs. 6.8%).

### 3.2. Association Between Hospital Surgical Volume and 30-Day Mortality

**Figure 2:**
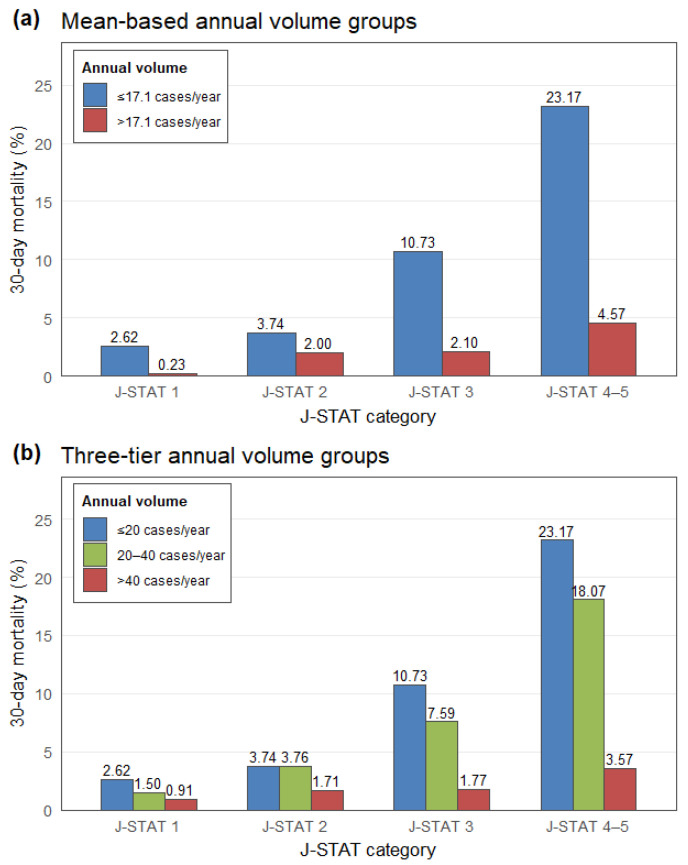
Thirty-Day Mortality According to J-STAT Category and Hospital Surgical Volume: (**a**) Mean-Based Annual Volume Groups (≤17.1 vs. >17.1 Cases/Year); (**b**) Three-Tier Annual Volume Groups (≤20, 21–40, and >40 Cases/Year).

Across all hospitals, 30-day mortality showed a strong inverse association with hospital annual surgical volume, and this pattern was consistent across all J-STAT mortality categories (Figure 2). In the mean-based comparison (≤17.1 vs. >17.1 cases/year), low-volume hospitals exhibited markedly higher observed mortality at every J-STAT level, with rates increasing from 2.62% in J-STAT 1 to 23.17% in J-STAT 4–5, whereas mortality in high-volume hospitals ranged from 0.23% to 4.57%. A similar gradient was observed when annual volume was categorized into low- (≤20 cases/year), medium- (21–40 cases/year), and high-volume (>40 cases/year) groups. Mortality declined steadily with increasing surgical volume; for example, in the highest-risk J-STAT 4–5 category, mortality was 23.17% in low-volume hospitals, 18.07% in medium-volume hospitals, and 3.57% in high-volume hospitals. These descriptive patterns were corroborated by regression analyses. In the mean-based model, hospitals performing ≤17.1 cases/year had substantially higher odds of 30-day mortality compared with those performing >17.1 cases/year (adjusted odds ratio [OR], 3.74; 95% CI, 3.09–4.53). In the three-tier model, the adjusted odds of mortality decreased monotonically across the low-, medium-, and high-volume groups, from 4.95 (95% CI, 3.98–5.95) to 4.13 (95% CI, 3.30–5.17) relative to the >40 cases/year reference group. A significant linear trend across ordered volume levels (*p* for trend <0.0001) indicated a clear dose–response relationship (Table 3). Taken together, these findings demonstrate that higher annual surgical volume is consistently associated with lower 30-day mortality, and that this inverse volume–outcome gradient becomes more pronounced as surgical complexity increases. In sensitivity analyses using 90-day mortality, the inverse association between annual surgical volume and mortality remained qualitatively unchanged (Table S3).

### 3.3. Regional Disparities in 30-Day Mortality and the Explanatory Role of Surgical Volume

**Figure 3:**
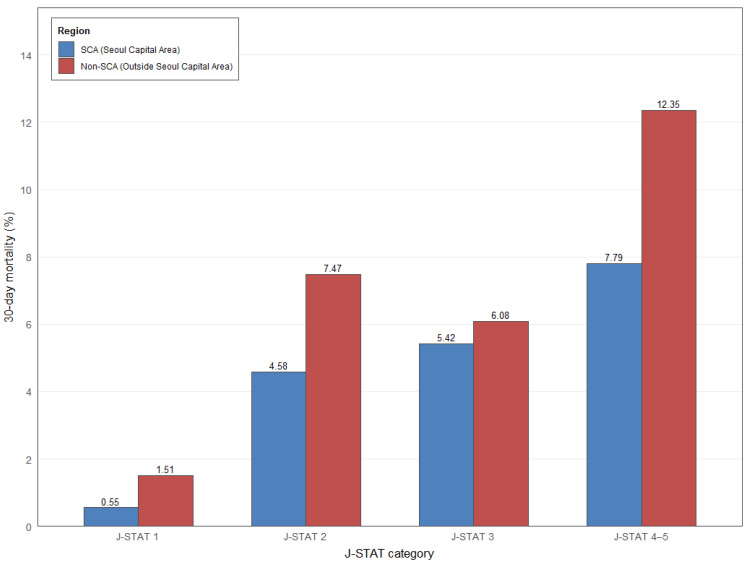
Thirty-Day Mortality by J-STAT Category According to Hospital Region (Seoul Capital Area vs. Non-Capital Area).

As shown in Figure 3, 30-day mortality was consistently higher in hospitals located outside the Seoul Capital Area across all J-STAT categories, with the largest gaps observed in higher-risk groups. This unadjusted regional difference was confirmed in the logistic regression analysis: in the univariable model, non-SCA hospitals had more than twice the odds of 30-day mortality compared with SCA hospitals (OR, 2.07; 95% CI, 1.76–2.43; *p* < 0.0001). The association persisted after adjustment for patient and hospital characteristics (Model 1: OR, 2.12; 95% CI, 1.80–2.49; *p* < 0.001). However, when annual surgical volume was additionally included in the model (Model 2), the regional disparity was no longer statistically significant (OR, 1.14; 95% CI, 0.95–1.37; *p* = 0.1660), indicating that the observed differences between SCA and non-SCA hospitals were largely explained by differences in surgical volume (Table 4). Sensitivity analyses using 90-day mortality yielded consistent findings, with substantial attenuation of the non-SCA vs. SCA difference after adjustment for annual surgical volume (Table S4). Notably, high-volume centers performing >40 cases/year accounted for most congenital heart surgeries, and within these centers, the regional gap in 30-day mortality was minimal (Supplementary Figure S1). This pattern was corroborated in the multivariable analysis, in which non-SCA hospitals did not differ significantly from SCA hospitals among high-volume centers (adjusted OR, 1.11; 95% CI, 0.84–1.46; *p* = 0.4632) (Supplementary Table S2), supporting that volume rather than geography primarily drives outcome differences. To determine whether regional differences in 30-day mortality reflected a true location effect or were attributable to hospital-level characteristics, we sequentially added capital-region status and surgical volume to multilevel models and evaluated changes in between-hospital variation. In the base model, the intraclass correlation coefficient (ICC) for 30-day mortality was 0.14 (95% CI, 0.07–0.21), indicating substantial between-hospital variation. Adding hospital location in the capital region produced only a modest reduction in ICC to 0.13 (95% CI, 0.07–0.20), explaining 7% of the variation, and this improvement was not statistically significant (*p* = 0.06). In contrast, further adjustment for hospital surgical volume markedly reduced the ICC to 0.06 (95% CI, 0.03–0.10), indicating that 57% of the between-hospital variation in 30-day mortality was explained by surgical volume. When considering only the change from the capital-adjusted model, surgical volume accounted for 54% of the remaining variation, and this improvement was highly significant (*p* < 0.0001) (Table 5).

## 4. Discussion

In this nationwide claims-based cohort study, we found a significant inverse association between hospital surgical volume and 30-day mortality. Notably, a substantial proportion of the mortality gap between SCA and non-SCA hospitals appeared to be attributable to differences in surgical volume rather than geography per se. Hospital surgical volume has been established in numerous international studies as a robust correlate of outcomes in congenital heart surgery [1,2,3,4,5,6,7,8,9,10,11]. Within the Donabedian framework, volume is a structural measure that is relatively easy and intuitive to quantify compared with many process and outcome metrics, and it has therefore been widely used in quality assessment and benchmarking programs as a proxy for institutional experience and capacity [21]. However, despite the face validity of hospital surgical volume as a quality indicator in congenital heart surgery, it has not been routinely incorporated into quality assessment or benchmarking frameworks for congenital heart surgery in Korea [22]. This omission may partly reflect the limited domestic evidence base on volume–outcome relationships. In this context, prior Korean evidence is limited. To the best of our knowledge, only one study has examined the association between hospital surgical volume and outcomes in congenital heart surgery. Using Korea Heart Foundation data from 2000 to 2014, the study offered useful descriptive insights but was limited by older data, limited case-mix adjustment, and the absence of regional information. Hospitals were grouped solely by volume without consideration of geographic context [17]. Our study addresses this evidence gap by providing contemporary, population-level estimates of the volume–outcome relationship using nationwide claims data with risk adjustment and explicit consideration of geographic context. The findings support surgical volume as a pragmatic structural indicator for quality surveillance in congenital heart surgery and highlight the need for volume-informed benchmarking frameworks in Korea. Beyond the Korean context, international expert groups have proposed structural models for organizing congenital heart surgery services, typically emphasizing minimum annual volume thresholds, multidisciplinary team requirements, and formal regional referral networks. The European Association of Cardio-Thoracic Surgery Congenital Heart Disease Committee, for example, outlined principles for the optimal structure of congenital heart surgery departments in Europe, including the consolidation of complex procedures in centers with sufficient case volume and robust quality assurance systems, while maintaining equitable access within defined catchment areas. These frameworks provide a useful conceptual benchmark for interpreting our findings and for considering how volume-informed standards might be adapted to the Korean health system [23]. Despite Korea’s relatively compact geography and nationally standardized health system, substantial regional gaps in outcomes persist, suggesting that structural features of care delivery may play an important role. Importantly, the distribution of surgical volume differed markedly by region: in the SCA, 84.0% of procedures were performed in high-volume centers (over 40 cases/year), whereas in non-SCA regions, only 55.9% were performed in such centers. This imbalance in access to high-volume care likely contributes to the observed regional disparities and supports regionalization strategies aimed at strengthening high-volume pediatric cardiac surgery capacity outside the capital region. Several countries have reported improved outcomes after regionalization strategies that concentrate complex pediatric cardiac surgery in higher volume centers. For example, Sweden reorganized pediatric cardiac surgery services by consolidating four centers into two, followed by a marked decline in national 30-day postoperative mortality despite increasing surgical complexity [24]. The United Kingdom illustrates another approach, in which a country with a larger population than South Korea delivers pediatric cardiac surgery through a relatively small number of nationally accredited centers within a coordinated congenital heart disease network [25]. In the United States, a nationwide simulation study similarly suggested that shifting care toward higher volume centers could reduce deaths with only modest increases in travel distance, and that limiting regionalization to only the highest risk cases would capture only part of the potential benefit [26]. Building on such international experiences, our findings suggest several pragmatic options for South Korea: formal designation of high-volume congenital heart surgery centers, structured referral pathways that prioritize transfer of complex neonatal and infant cases to these centers, and transparent, risk-adjusted public reporting of outcomes to support accountability and patient choice. In addition, given the highly distributed provision of congenital heart surgery across multiple programs in non-SCA regions—and the practical constraints of sustaining multidisciplinary teams and specialized workforce under limited local resources—our results support a more coordinated configuration, such as consolidation of the most complex neonatal/infant surgeries into designated regional hubs and strengthened network-based care (including timely transfer pathways) for surrounding hospitals. Implementing these changes would require coordinated action by government, professional societies, and patient or parent advocacy groups, but could provide a concrete roadmap for moving from a highly distributed system toward a more planned and equitable national configuration. Several limitations merit consideration. First, although our findings are consistent with international evidence suggesting that regionalization and concentration of care in high-volume centers can improve outcomes, this study was observational and not designed to directly evaluate the causal impact of regionalization policies. We were therefore unable to assess potential trade-offs associated with centralization, such as increased travel distance, delays in access, or family burden, which have been highlighted in prior modeling studies from other countries. Second, surgical volume was measured at the hospital level and may not fully capture surgeon-specific experience, team composition, or institutional processes that also contribute to outcomes. Third, despite extensive risk adjustment, residual confounding by unmeasured clinical factors cannot be completely excluded. Finally, our classification of hospital surgical volume should be interpreted in context. International consensus statements often propose high absolute thresholds for defining comprehensive congenital heart surgery centers (e.g., ≥200 cases/year), but such criteria are difficult to transfer directly across health systems with different workforce structures and patterns of case distribution. In Korea, only one of the 91 hospitals met the ≥200 cases/year threshold, and even smaller thresholds (e.g., >40 cases/year in our study) may not be directly comparable to “high-volume” definitions in other countries because the meaning of a given annual volume depends on factors such as the number of congenital cardiac surgeons and team staffing, how cases are shared across providers, and institutional organization. We considered higher absolute cutoffs, but only one hospital met the ≥200 cases/year threshold, precluding meaningful stratification. To reflect the observed national distribution, we therefore adopted empirically informed cutoffs. Importantly, the inverse volume–outcome relationship was robust across alternative classifications, and similar attenuation of regional disparities was observed when annual volume was modeled as a continuous variable, indicating that our conclusions were not artifacts of a specific categorization scheme.

## 5. Conclusions

In this nationwide, population-based cohort study, we demonstrated a strong inverse association between hospital surgical volume and short-term mortality in congenital heart surgery in Korea. A substantial proportion of the observed regional disparities between SCA and non-SCA hospitals appeared to be explained by differences in surgical volume rather than geography alone. These findings suggest that unequal access to high-volume centers remains a key structural driver of outcome inequities, despite Korea’s compact geography and standardized health system. Taken together, our results support hospital surgical volume as a pragmatic and informative indicator for quality surveillance and highlight the potential value of volume-informed regionalization strategies to improve outcomes and reduce regional disparities in pediatric cardiac surgery in Korea.

## Figures and Tables

**Table 1:** Patient characteristics by hospital region (Seoul Capital Area vs. Non-Capital Area), 2002–2021.

Characteristic	Total (n = 31,150)	Seoul Capital Area Hospitals (n = 22,995)	Non-Capital Area Hospitals (n = 8155)	*p* Value
**Male Sex**	16,337 (52.4)	12,101 (52.6)	4236 (51.9)	0.2901
**Age < 1 year**	20,042 (64.3)	14,520 (63.1)	5522 (67.7)	<0.0001
**Gestational age < 37 weeks**	4231 (13.6)	2857 (12.4)	1374 (16.9)	<0.0001
**Birth weight < 2500 g**	3188 (10.2)	2115 (9.2)	1073 (13.2)	<0.0001
**J-STAT mortality category**				
J-STAT 1	10,779 (34.6)	7994 (34.8)	2785 (34.2)	<0.0001
J-STAT 2	12,052 (38.7)	8358 (36.4)	3694 (45.3)	
J-STAT 3	5829 (18.7)	4744 (20.6)	1085 (13.3)	
J-STAT 4	2404 (7.7)	1831 (8.0)	573 (7.0)	
J-STAT 5	86 (0.3)	68 (0.3)	18 (0.2)	
**Patients treated at hospitals categorized by average annual volume (mean-based)**				
≤17.1 cases/year	4622 (14.8)	2211 (9.6)	2411 (29.6)	<0.0001
>17.1 cases/year	26,528 (85.2)	20,784 (90.4)	5744 (70.4)	
**Patients treated at hospitals categorized by average annual volume (3-tier)**				
≤20 cases/year	4622 (14.8)	2211 (9.6)	2411 (29.6)	<0.0001
21–40 cases/year	2662 (8.6)	1482 (6.4)	1180 (14.5)	
>40 cases/year	23,866 (76.6)	19,302 (84.0)	4564 (55.9)	

Values are presented as n (%). *p* values were calculated using the chi-square test.

**Table 2:** Hospital characteristics by hospital region (Seoul Capital Area vs. Non-Capital Area), 2002–2021.

Characteristic	Total (n = 91 Hospitals)	Seoul Capital Area Hospitals (n = 47 Hospitals)	Non-Capital Area Hospitals (n = 44 Hospitals)
**Cumulative CHS case volume per hospital during 20 years, mean (range)**	342 (1–6283)	489 (1–6283)	185 (1–2284)
**Hospitals by average annual surgical volume (mean-based)**			
≤17.1 cases/year	78 (85.7)	39 (83.0)	39 (88.6)
>17.1 cases/year	13 (14.3)	8 (17.0)	5 (11.4)
**Hospitals by average annual surgical volume (3-tier)**			
≤20 cases/year	78 (85.7)	39 (83)	39 (88.6)
21–40 cases/year	5 (5.5)	3 (6.4)	2 (4.5)
>40 cases/year	8 (8.8)	5 (10.6)	3 (6.8)

n (%) unless otherwise noted; Column headers indicate the number of hospitals in each group. Row 1 summarizes cumulative CHS case volume per hospital during 2002–2021 (mean and range). Subsequent rows report the number (%) of hospitals by average annual volume categories (cases/year).

**Table 3:** Thirty-Day Mortality According to Annual Surgical Volume Categories.

Annual Surgical Volume Category	Univariable Model	Multivariable Model *
	Unadjusted Odds Ratio (95% CI)	Adjusted Odds Ratio (95% CI)
**Mean-based annual volume groups**		
≤17.1 cases/year	2.70 (2.27–3.21)	3.74 (3.09–4.53)
>17.1 cases/year	1.00 (reference)	1.00 (reference)
**Three-tier annual volume groups**		
≤20 cases/year	3.42 (2.74–4.25)	4.95 (3.98–5.95)
21–40 cases/year	3.34 (2.78–4.00)	4.13 (3.30–5.17)
>40 cases/year	1.00 (reference)	1.00 (reference)
***p* for trend**	<0.0001	<0.0001

CI denotes confidence interval. * Multivariable models were adjusted for J-STAT category, sex, hospital type, prematurity, low birth weight, and age at surgery.

**Table 4:** Thirty-Day Mortality According to Hospital Region.

Hospital Region	Univariable Model	*p* Value	Multivariable Model 1 *	*p* Value	Multivariable Model 2 ^†^	*p* Value
	Unadjusted Odds Ratio (95% CI)		Adjusted Odds Ratio (95% CI)		Adjusted Odds Ratio (95% CI)	
non-SCA	2.07 (1.76–2.43)	<0.0001	2.12 (1.80–2.49)	<0.0001	1.14 (0.95–1.37)	0.1660
SCA	1.00 (reference)		1.00 (reference)		1.00 (reference)	

CI denotes confidence interval; SCA denotes Seoul Capital Area; non-SCA denotes hospitals located outside the Seoul Capital Area; * Multivariable Model 1 adjusted for J-STAT category, sex, hospital type, prematurity, low birth weight, and age; ^†^ Multivariable Model 2 adjusted for all variables in Model 1 plus annual surgical volume.

**Table 5:** Changes in Between-Hospital Variation in 30-Day Mortality After Adding Hospital-Level Variables.

Model	ICC (95% CI)	% Explained vs. Base	% Explained vs. Capital	*p* Value
Base	0.14 (0.07–0.21)	Reference	-	-
+Capital	0.13 (0.07–0.20)	7%	-	0.06
+Capital + Volume	0.06 (0.03–0.10)	57%	54%	<0.0001

The intraclass correlation coefficient (ICC) indicates the proportion of total variation in the outcome attributable to hospital-level clustering. Models were sequentially adjusted by adding hospital-level characteristics: the base model included patient-level covariates only; the “+Capital” model added hospital location in the capital region; and the “+Capital + Volume” model further included hospital surgical volume. *p* values were obtained from likelihood ratio tests comparing nested multilevel models.

## Data Availability

The data used in this study were obtained from the National Health Insurance Service (NHIS) of South Korea under a data use agreement; therefore, restrictions apply to their availability. The data are not publicly available and can be accessed only through an application and approval process administered by the NHIS.

## Supplementary Materials

The following supporting information can be downloaded at: https://www.mdpi.com/article/10.3390/medicina62020355/s1, Figure S1: Thirty-Day Mortality by J-STAT Category According to Annual Hospital Surgical Volume and Hospital Region (Seoul Capital Area vs. Non-Seoul Capital Area): (a) Low-volume centers (≤20 cases/year); (b) Medium-volume centers (21–40 cases/year); (c) High-volume centers (>40 cases/year); Table S1: Congenital heart surgery procedures, NHIS reimbursement codes, and mortality categories; Table S2: Thirty-Day Mortality According to Hospital Region, Stratified by Annual Surgical Volume; Table S3: Ninety-Day Mortality According to Annual Surgical Volume Categories; Table S4: Ninety-Day Mortality According to Hospital Region.

## Abbreviations

The following abbreviations are used in this manuscript:

CHS	Congenital Heart Surgery
SCA	Seoul Capital Area
NHIS	National Health Insurance Service
KCD	Korean Standard Classification of Diseases
ICD	International Classification of Diseases
J-STAT	Japanese Surgical Risk Category
STAT	Society of Thoracic Surgeons-European Association for Cardio-Thoracic Surgery mortality category
STS	Society of Thoracic Surgeons
OR	Odds Ratio
CI	Confidence Interval
ICC	Intraclass Correlation Coefficient
