# Supplementary material for: Hospital Surgical Volume and Regional Disparities in Congenital Heart Surgery Outcomes: Analysis of Korean National Health Insurance Claims Data, 2002–2021
Source: Medicina (Kaunas). 2026 Feb 11;62(2):355. doi: 10.3390/medicina62020355 (PMC12943574; doi:10.3390/medicina62020355)
Supplement: Supplementary file 1 [file medicina-62-00355-s001.zip › medicina-4099562-supplementary.pdf]

Table S1. Congenital heart surgery procedures, NHIS reimbursement codes, and mortality categories

Procedure name	NHIS reimbursement codes	Mortality category
Operation for atrial septal defect	O1710, O1711	1
Operation for ventricular septal defect – Congenital	O1721, O1723	1
Correction of aortic coarctation	O1680	2
Closure of patent ductus arteriosus – Ligation	O1671	2
Closure of patent ductus arteriosus – Division	O1672	2
Shunt procedure	O1701	2
Surgery for pulmonary stenosis	O1750	2
Operation for atrial septal defect and pulmonary valvular stenosis	O1770	2
Valvuloplasty – Aortic valve	O1783	2
Operation for ventricular septal defect and pulmonary valvular stenosis	O1810	2
Repair of endocardial cushion defect: partial	O1821	2
Repair of endocardial cushion defects	O1822	2
Left ventricular outflow tract augmentation	O1825	2
Right ventricular outflow tract reconstruction	O1826	2
Repair of ruptured aneurysm of the sinus Valsalva	O1840	2
Pulmonary artery banding	O1703	3
Atrial septostomy	O1705	3
Valvuloplasty – Tricuspid valve	O1781	3
Valvuloplasty – Mitral valve	O1782	3
Valve replacement – Tricuspid valve	O1791	3
Valve replacement – Mitral valve	O1792	3
Valve replacement – Aortic valve	O1793	3
Total correction of tetralogy of Fallot	O1800	3
Left and right pulmonary artery reconstructions	O1861	3
Functional correction of single ventricle – Glenn operation	O1873	3
Repair of complicated congenital heart diseases – Others	O1852	4
Functional correction of single ventricle – Fontan operation	O1874	4
Repair of total anomalous pulmonary venous return	O1878	4
Repair of transposition of the great arteries	O1879	4
Rastelli operation	O1875	4
Repair of complicated congenital heart diseases – Highly complicated (Norwood, Nikaidoh, Damus–Kaye–Stansel, MAPCA unifocalization, truncus arteriosus correction, double switch)	O1851	5

Table S2. Thirty-Day Mortality According to Hospital Region, Stratified by Annual Surgical Volume

**Annual surgical volume stratum / Hospital region**	**Multivariable Model** Adjusted Odds Ratio (95% CI)	***P* value**
**≤ 20 cases/year**		
Non-SCA	1.26 (0.94-1.69)	0.1291
SCA	1.00 (reference)	
**21-40 cases/year**		
Non-SCA	3.04 (1.99-4.65)	<0.0001
SCA	1.00 (reference)	
**>40 cases/year**		
Non-SCA	1.11 (0.84-1.46)	0.4632
SCA	1.00 (reference)	

CI denotes confidence interval.
**** Multivariable models were adjusted for J-STAT category, sex, hospital type, prematurity, low birth weight, and age at surgery.***

Table S3. Ninety-Day Mortality According to Annual Surgical Volume Categories

Annual Surgical Volume Category	Univariable Model	Multivariable Model
	Unadjusted Odds Ratio (95% CI)	Adjusted Odds Ratio (95% CI)
**Mean-based annual volume groups**		
≤17.1 cases/year	2.31 (2.03–2.63)	2.74 (2.36–3.17)
>17.1 cases/year	1.00 (reference)	1.00 (reference)
**Three-tier annual volume groups**		
≤ 20 cases/year	2.58 (2.26–2.95)	3.19 (2.75–3.72)
21–40 cases/year	2.23 (1.88–2.65)	2.54 (2.13–3.04)
>40 cases/year	1.00 (reference)	1.00 (reference)
**P for trend**	<0.0001	<0.0001

CI denotes confidence interval. * Multivariable models were adjusted for J-STAT category, sex, hospital type, prematurity, low birth weight, and age at surgery.

Table S4. Ninety-Day Mortality According to Hospital Region

Hospital Region	**Univariable Model** Unadjusted Odds Ratio (95% CI)	***P* value**	**Multivariable Model 1** Adjusted Odds Ratio (95% CI)	***P* value**	**Multivariable Model 2** Adjusted Odds Ratio (95% CI)	***P* value**
Non-SCA	1.72 (1.53–1.94)	<0.0001	1.73 (1.53–1.96)	<0.0001	1.12 (0.99–1.26)	0.0570
SCA	1.00 (reference)		1.00 (reference)		1.00 (reference)	

CI denotes confidence interval; SCA denotes Seoul Capital Area; Non-SCA denotes hospitals located outside the Seoul Capital Area; * Multivariable Model 1 adjusted for J-STAT category, sex, hospital type, prematurity, low birth weight, and age; † Multivariable Model 2 adjusted for all variables in Model 1 plus annual surgical volume.

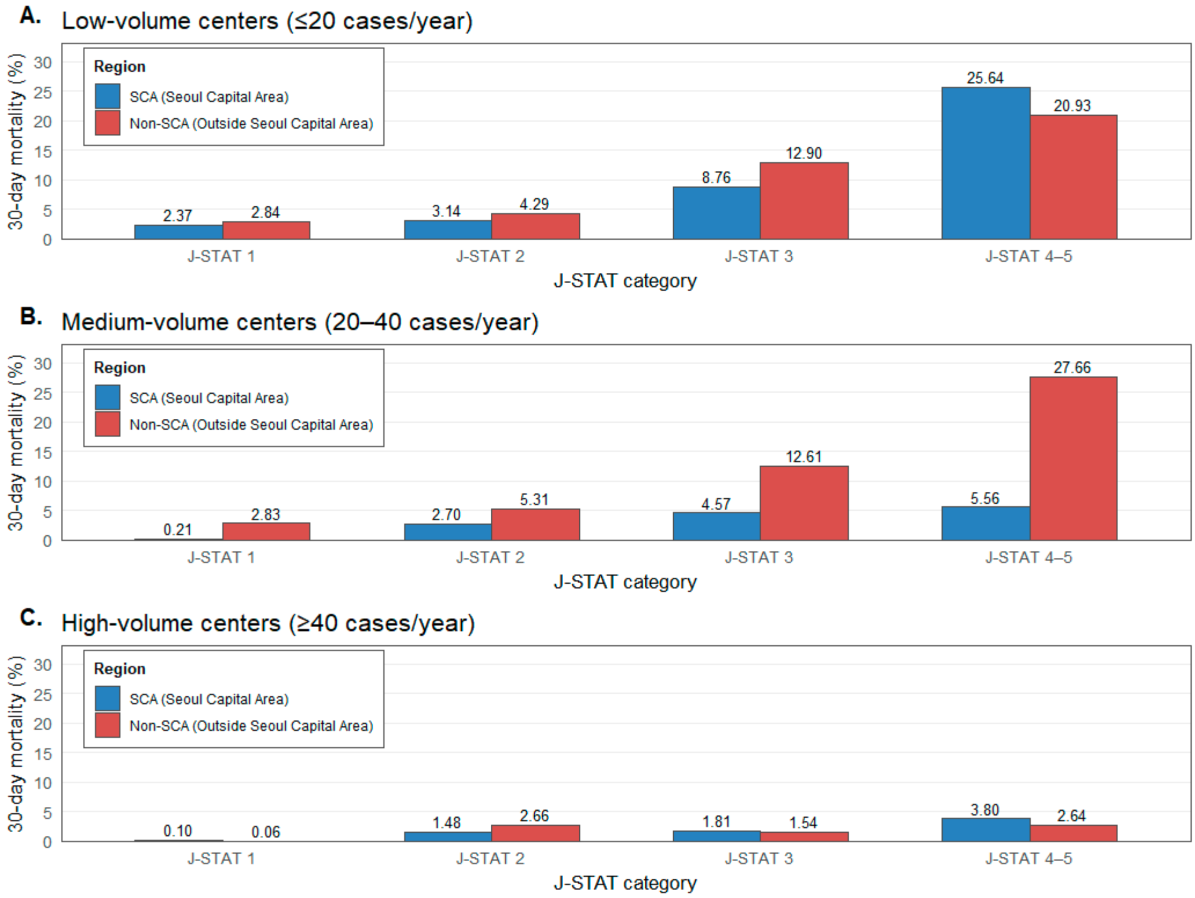

Figure S1. Thirty-Day Mortality by J-STAT Category According to Annual Hospital Surgical V olume and Hospital Region (Seoul Capital Area vs. Non-Seoul Capital Area): (a) Low-volume centers (≤20 cases/year); (b) Medium-volume centers (21–40 cases/year); (c) High-volume centers (>40 cases/year)
